# Assessment of the left ventricular function in normotensive prediabetics: a tissue Doppler echocardiography study

**DOI:** 10.1590/2359-3997000000136

**Published:** 2015-01-01

**Authors:** Murat Akçay, Abdullah N. Aslan, Hacı A. Kasapkara, Hüseyin Ayhan, Tahir Durmaz, Telat Keleş, Engin Bozkurt

**Affiliations:** 1 Yildirim Beyazit University Faculty of Medicine Department of Cardiology Ankara Turkey Yildirim Beyazit University Faculty of Medicine, Department of Cardiology, Ankara, Turkey; 2 Atatürk Education and Research Hospital Department of Cardiology Ankara Turkey Atatürk Education and Research Hospital, Department of Cardiology, Ankara, Turkey

**Keywords:** Prediabetes, left ventricular function, tissue Doppler echocardiography

## Abstract

**Objective:**

Several studies have shown that left ventricular (LV) dysfunction is increased in individuals with diabetes. However, there are scarce data about LV function in prediabetics. This study assessed the early changes in LV diastolic and systolic myocardial function in normotensive prediabetics using tissue Doppler echocardiography (TDE).

**Subjects and methods:**

We evaluated 94 patients with prediabetes (mean age of 50.8 ± 6.9 years, 78 female) without known cardiovascular diseases and 70 healthy volunteers with similar demographic characteristics. Systolic and diastolic function of the left ventricle was evaluated with transthoracic echocardiography according to the latest consensus recommendations including TDE.

**Results:**

The mean results of septal and lateral parts of the mitral annulus Pulsed wave TDE showed that myocardial systolic wave (Sm), myocardial early diastolic wave (Em) and Em to atrial peak velocity (Am) ratio were significantly lower whereas early diastolic peak flow velocity (E) to Em ratio, myocardial isovolumetric relaxation time (IVRTm), myocardial isovolumetric contraction time (IVCTm) and myocardial performance index (MPI) values were significantly higher in patients with prediabetes (preDM). In addition, mean left atrium (LA) diameter measured with M-mode echocardiography was significantly higher in prediabetics than controls.

**Conclusion:**

PreDM is associated with subclinical LV systolic and diastolic dysfunction as evaluated by TDE.

## INTRODUCTION

Type 2 diabetes affects more than 8% of the United States population (1). The onset of type 2 diabetes is gradual, with most patients progressing through a state of prediabetes which is defined as one or more of the following: impaired fasting glucose (IFG) (plasma glucose of 100 to 125 mg/dL), impaired glucose tolerance (IGT) (plasma glucose of 140 to 199 mg/dL 2 hours after an oral load of 75 g dextrose) or HbA1c 5.7% to 6.4% (2). Although individuals can spend years in prediabetic stage, an expert American Diabetes Association (ADA) panel estimated that up to 70% of individuals with prediabetes (preDM) will eventually progress to type 2 diabetes (3). For this reason, preDM is considered a substantial risk factor for diabetes and is associated with microvascular complications and cardiovascular disease in a way similar to diabetes mellitus (DM) (4,5). It is associated with an approximately 20% increased risk of developing cardiovascular disease compared with normoglycemic subjects. The underlying pathophysiological disturbances of excess risk from prediabetes are presumed to be the same as those from diabetes such as insulin resistance and impaired beta cell function (6). Although DM is known to be related with left ventricular (LV) dysfunction even after hypertension (HT) and coronary artery disease (CAD) are excluded, the association between preDM and LV function has not been comprehensively investigated.

Therefore, in the present study, we aimed to investigate the possible impact of prediabetes on both LV systolic and diastolic function using conventional echocardiographic techniques including tissue Doppler echocardiography (TDE).

## SUBJECTS AND METHODS

### Study population

The overall study population consisted of 164 subjects: 94 subjects with preDM (52 patients with IFG, 23 patients with IGT and 19 patients with IFG+IGT) and 70 normoglycemic subjects with normal glucose tolerance. In each subject, fasting plasma glucose (FPG) and HbA1c levels were measured. The diagnosis of PreDM was ensured according to the FPG level and/or oral glucose tolerance test (OGTT) results confirmed by repeating the tests on another day and/or measuring the HbA1c level based on American Diabetes Association (ADA) guidelines (1,2). Subjects with a FPG level of 100 to 125 mg/dL were convinced to have IFG. In patients with a normal or IFG level, OGTT was performed. OGTT measures the level of blood glucose after a person fasts for at least 8 hours and two hours after the subject drinks a beverage containing 75 grams of glucose dissolved in water. If the 2-hour blood glucose level is between 140 and 199 mg/dL, then the person is considered to have IGT. In addition, individuals with HbA1c levels between 5.7 to 6.5% were diagnosed as having preDM. As a control group, we have studied 70 healthy subjects without preDM and overt cardiovascular diseases.

Exclusion criteria were to have structural heart disease, hypertension, diabetes mellitus, pulmonary disease, neoplastic and chronic systemic diseases, age over 60 years or under 18 years. Informed content was taken from each subject participated into the study. Approval of the Local Ethics Board was received from our hospital for the study protocol.

### Echocardiographic examination

All echocardiographic examinations were carried out using 2.5–3.5 MHz transducer with the Vingmed System 7 (Vivid 7, GE, Horten, Norway) by two experienced cardiologists performing the measurements blinded to preDM status. Then, data from these cardiologists were averaged. Inter-observer variability was satisfactory. The concordance correlation coefficients demonstrated close agreement between the two observers, from 0.83 to 0.96. Two-dimensional, M-mode and subsequent TDE and quantitative analysis were conducted on parasternal long axis, short axis and apical four-chamber images according to the data provided by the American Society of Echocardiography (7).

Left ventricular systolic and diastolic functions were analyzed using standard two-dimensional (2D) echocardiography, M-mode echocardiography, pulsed wave (PW) echocardiography and TDE. Diameter of the left atrium (LA), thickness of the interventricular septum (IVS), thickness of the posterior wall (PoW), left ventricular end-diastolic diameter (LVEDD) and left ventricular end-systolic diameter (LVESD) were obtained from the M-mode echocardiographic tracing under the guidance of 2D imaging. LV ejection fraction was calculated by the modified Simpson’s method. The pulsed Doppler sample volume was positioned at the mitral leaflet tips. Early diastolic peak flow velocity (E), late diastolic peak flow velocity (A), E-wave deceleration time (DT), isovolumetric contraction time (IVCT), ejection time (ET) and isovolumetric relaxation time (IVRT) were measured by transmitral Doppler imaging. E/A ratio was calculated. Myocardial performance index (MPI) was calculated by summing of IVRT and IVCT and dividing by ET.

Tissue Doppler echocardiography (TDE) was used to obtain LV myocardial velocities in the apical four-chamber view with a 5 mm sample volume on the medial and lateral corner of the mitral annulus and the mean results of these parameters were obtained. TDE program was set to the PW Doppler mode. Filters were set to minimize high-frequency signals, and Nyquist limit was adjusted to a velocity range of -15 to 20 cm/s. The following measurements were obtained in each region as: myocardial systolic (Sm) wave, myocardial early diastolic wave (Em) and atrial peak velocity (Am), Em/Am ratio, myocardial isovolumetric relaxation time (IVRTm), myocardial isovolumetric contraction time (IVCTm), myocardial ejection time (ETm) and IVCTm/ETm ratio. MPI was calculated by summing IVCTm and IVRTm and dividing by ETm value. In addition to these parameters, E/Em ratio, a reliable index of LV filling pressures was measured. All diastolic parameters were measured in three consecutive cardiac cycles and averaged.

### Statistical analysis

The analysis were performed using the SPSS 17.0 Software package program (SPSS Inc., Chicago, IL, USA). Whether the distribution of continuous variables is normal or not was analyzed with the Shapiro–Wilk test. Data are expressed as mean ± SD for continuous variables, and as number of observations and percentage (%) for categorical variables. Significance of differences in terms of features obtained by measurements from control and patient groups were analyzed with Student’s t test or Mann-Whitney U-test. Pearson’s Chi-square test was used for comparison of categorical variables. A P-value less than 0.05 was considered statistically significant.

## RESULTS

### Clinical characteristics of the subjects

The baseline characteristics of the study population were presented in Table 1. Age, gender, body mass index (BMI), systolic blood pressure (SBP), diastolic blood pressure (DBP), heart rate, smoking rate, total cholesterol, low-density lipoprotein (LDL) cholesterol, high-density lipoprotein (HDL) cholesterol and triglyceride levels were similar among the groups. However fasting blood glucose level (107.0 ± 10.8 mg/dL *vs* 88.8 ± 6.4 mg/dL, respectively, p < 0.001) and HbA1c level (5.98 ± 0.22% *vs* 5.09 ± 0.22%, respectively, p < 0.001) were significantly higher in preDM group compared to controls.

**Table 1:** The clinical and demographic characteristics of the subjects

Variables	Patients (n = 94)	Controls (n = 70)	P-value
Age (years)	50.8 ± 6.9	50.3 ± 7.7	0.089
Gender (M/F)	16/78	12/58	0.270
BMI (kg/m^2^)	29.5 ± 4.9	29.1 ± 3.8	0.475
SBP (mmHg)	122.6 ± 12.3	120.1 ± 11.3	0.123
DBP (mmHg)	74.5 ± 8.8	72.9 ± 8.2	0.239
Pulse (beats/min)	76.7 ± 11.8	76.3 ± 10.1	0.811
Current smokers (%)	14	16	0.377
FBG (mg/dL)	107.0 ± 10.8	88.8 ± 6.4	< 0.001
HbA1C (%)	5.98 ± 0.22	5.09 ± 0.22	< 0.001
Total cholesterol (mg/dL)	194.9 ± 35.4	187.8 ± 36.9	0.337
LDL cholesterol (mg/dL)	115.1 ± 25.0	118.2 ± 30.6	0.586
HDL cholesterol (mg/dL)	47.0 ± 10.1	46.0 ± 9.5	0.617
Triglyceride (mg/dL)	145.0 ± 79.7	126.4 ± 70.0	0.223

M/F: male/female; BMI: body mass index; SBP: systolic blood pressure; DBP: diastolic blood pressure; FBG: fasting blood glucose; HbA1C: glycosylated hemoglobin; LDL: low-density lipoprotein; HDL: high-density lipoprotein.

### Analysis of the echocardiographic measurements

The conventional echocardiographic results of the subjects were presented in Table 2. Accordingly, LV end-diastolic and end-systolic diameters, thickness of IVS and PoW and LV ejection fraction were similar among the groups. However, LA diameter was significantly higher in preDM group than in controls (35.6 ± 3.8 mm *vs* 33.9 ± 4.4 mm, respectively, p = 0.010). However, when subgroup analysis were performed, LA diameter was not significantly higher in IFG+IGT group compared to controls (34.6 ± 3.7 mm *vs* 33.9 ± 4.4 mm, p = 0.508). Pulse-wave echocardiographic measurements of the subjects were demonstrated in Table 3. According to these results, A wave, IVRT, IVCT/ET ratio and DT were significantly longer whereas E wave and E/A ratio were significantly lower in preDM group than in controls. Subgroup analysis also showed similar results except for A wave that was similar between the controls and IFG only group.

**Table 2:** Conventional echocardiographic results of the subjects

Variables	Controls (n = 70)	Patients (n = 94) (p value)	IFG only (n = 52) (p value)	IGT only (n = 23) (p value)	IFG and IGT (n = 19) (p value)
LVEDD (mm)	44.5 ± 3.4	43.9 ± 3.4 (0.293)	43.9 ± 3.4 (0.344)	44.9 ± 2.9 (0.606)	42.7 ± 3.9 (0.067)
LVESD (mm)	25.6 ± 2.56	24.9 ± 3.1 (0.116)	24.6 ± 2.7 (0.031)	25.8 ± 3.2 (0.833)	24.7 ± 3.9 (0.239)
LA (mm)	33.9 ± 4.4	35.6 ± 3.8 (0.010)	35.6 ± 4.1 (0.033)	36.3 ± 3.0 (0.018)	34.6 ± 3.7 (0.508)
IVS (mm)	9.0 ± 1.0	9.3 ± 1.6 (0.191)	9.1 ± 1.4 (0.433)	9.3 ± 2.3 (0.288)	9.5 ± 1.1 (0.064)
PoW (mm)	8.9 ± 1.1	9.1 ± 1.2 (0.067)	9.3 ± 1.3 (0.041)	9.4 ± 2.3 (0.098)	9.5 ± 1.0 (0.022)
LVEF (%)	65.4 ± 3.7	65.4 ± 2.3 (0.901)	65.8 ± 1.5 (0.477)	64.9 ± 3.6 (0.559)	64.7 ± 2.2 (0.415)

LVEDD: left ventricular end-diastolic diameter; LVESD: left ventricular end-sistolic diameter; LA: left atrium; IVS: interventricular septum; PoW: posterior wall; LVEF: left ventricular ejection fraction.

**Table 3:** Pulse-wave echocardiographic measurements of the subjects

Variables	Controls (n = 70)	Patients (n = 94) (p value)	IFG only (n = 52) (p value)	IGT only (n = 23) (p value)	IFG and IGT (n = 19) (p value)
E (m/s)	0.9 ± 0.13	0.7 ± 0.16 (< 0.001)	0.7 ± 0.16 (< 0.001)	0.7 ± 0.16 (< 0.001)	0.6 ± 0.15 (< 0.001)
A (m/s)	0.74 ± 0.09	0.8 ± 0.21 (0.013)	0.7 ± 0.21 (0.130)	0.81 ± 0.20 (0.028)	0.87 ± 0.22 (< 0.001)
E/A ratio	1.2 ± 0.21	0.9 ± 0.35 (< 0.001)	1.0 ± 0.36 (< 0.001)	0.95 ± 0.34 (< 0.001)	0.79 ± 0.32 (< 0.001)
DT (ms)	167 ± 27.8	216 ± 44.0 (< 0.001)	219.0 ± 47.0 (< 0.001)	218.6 ± 47.5 (< 0.001)	207 ± 29.2 (< 0.001)
IVCT (ms)	68.9 ± 12.0	66.2 ± 14.0 (0.204)	63.3 ± 12.6 (0.015)	69.3 ± 14.8 (0.888)	70.4 ± 15.4 (0.650)
ET (ms)	284 ± 29.7	288 ± 28.1 (0.443)	286.0 ± 30.5 (0.831)	283.6 ± 25.9 (0.863)	300.4 ± 21.0 (0.036)
IVCT/ET ratio	0.23 ± 0.04	0.24± 0.4 (0.542)	0.22 ± 0.05 (0.641)	0.24 ± 0.05 (0.796)	0.23 ± 0.05 (0.524)
IVRT (ms)	69.2 ± 14.2	85.2 ± 12.7 (< 0.001)	83.7 ± 12.8 (< 0.001)	85.9 ± 14.8 (< 0.001)	88.6 ± 8.8 (< 0.001)
MPI	0.48 ± 0.08	0.51 ± 0.09 (0.051)	0.51 ± 0.06 (0.240)	0.52 ± 0.14 (0.120)	0.52 ± 0.07 (0.036)

E: early diastolic wave; A: late diastolic wave; DT: deceleration time; IVCT: isovolumetric contraction time; ET: ejection time; IVRT: isovolumetric relaxation time; MPI: myocardial performance index.

The mean results of the lateral and septal wall Pulse-wave tissue Doppler echocardiographic results of the subjects were presented in Table 4. Accordingly, it was found that Sm, Em and Em/Am ratio were significantly lower but IVRTm, IVCTm, IVCTm/ETm, E/Em ratio and MPI values were significantly higher in patient group. Subgroup analysis was also similar.

**Table 4:** The mean results of lateral and septal wall Pulsed wave tissue Doppler echocardiography measurements

Variables	Controls (n = 70)	Patients (n = 94) (p value)	IFG only (n = 52) (p value)	IGT only (n = 23) (p value)	IFG and IGT (n= 19) (p value)
Sm (cm/s)	9.7 ± 1.6	8.3 ± 1.6 (< 0.001)	8.2 ± 1.6 (< 0.001)	8.3 ± 1.7 (0.001)	8.4 ± 1.5 (0.003)
Em (cm/s)	12.9 ± 2.5	7.9 ± 1.8 (< 0.001)	8.2 ± 1.8 (< 0.001)	7.8 ± 1.9 (< 0.001)	7.3 ± 1.4 (< 0.001)
Am (cm/s)	10.1 ± 1.7	10.0 ± 1.8 (0.725)	10.0 ± 1.8 (0.574)	9.9 ± 1.9 (0.595)	10.4 ± 1.6 (0.517)
Em/Am ratio	1.3 ± 0.35	0.8 ± 0.28 (< 0.001)	0.8 ± 0.29 (< 0.001)	0.8 ± 0.29 (< 0.001)	0.7 ± 0.19 (< 0.001)
E/Em ratio	0.06 ± 0.02	0.08 ± 0.02 (< 0.001)	0.09 ± 0.02 (< 0.001)	0.09 ± 0.03 (< 0.001)	0.09 ± 0.02 (< 0.001)
IVCTm (ms)	66.4 ± 12.2	84.7 ± 9.5 (< 0.001)	83.3 ± 10.3 (< 0.001)	88.2 ± 8.2 (< 0.001)	84.2 ± 7.8 (< 0.001)
ETm (ms)	292.1 ± 25.5	285.0 ± 30.3 (0.117)	288.3 ± 27.0 (0.431)	282.0 ± 35.2 (0.139)	279.8 ± 33.2 (0.084)
IVCTm/ETm ratio	0.22 ± 0.03	0.30 ± 0.05 (< 0.001)	0.29 ± 0.05 (< 0.001)	0.31 ± 0.05 (< 0.001)	0.30 ± 0.04 (< 0.001)
IVRTm (ms)	65.9 ± 11.8	95.4 ± 16.1 (< 0.001)	97.1 ± 16.8 (< 0.001)	94.0 ± 16.1 (< 0.001)	92.5 ± 13.9 (< 0.001)
MPI	0.45 ± 0.06	0.63 ± 0.08 (< 0.001)	0.63 ± 0.08 (< 0.001)	0.65 ± 0.08 (< 0.001)	0.63 ± 0.07 (< 0.001)

Sm: systolic myocardial wave; Em: early diastolic myocardial wave; Am: late diastolic myocardial wave; E: early diastolic wave; IVCTm: myocardial isovolumetric contraction time; ETm: myocardial ejection time; IVRTm: myocardial isovolumetric relaxation time; MPI: myocardial performance index.

## DISCUSSION

In this study, we have found that patients with preDM have both impaired LV systolic and diastolic function. Because of the fact that we excluded patients with HT, CAD and other chronic systemic illnesses, LV dysfunction observed in patient group probably depends directly on impaired glucose metabolism seen in preDM. Hyperglycemia has been demonstrated to cause the formation of advanced glycosylation end products (AGE) and it enhances progressive loss of cardiomyocytes and increases fibrosis resulting from oxidative stress and inflammation. In diabetic heart, ventricular hypertrophy, metabolic abnormalities, extracellular matrix remodelling, fibrosis, vascular changes, insulin resistance, oxidative stress and apoptosis are the most important changes that may affect the myocardial function. Therefore, it has been assumed that prediabetic patients may have decreased LV function due to prolonged exposure to high glucose levels (8,9). A recent cross-sectional study reported that the prevalence of diastolic dysfunction increased in prediabetic patients and the severity of diastolic dysfunction was associated with the degree of impairment of glucose metabolism along with the whole spectrum of metabolic states (9). Hyperglycemia may also stimulate apoptotic cell death and myocyte necrosis (10) and results in myocardial cell loss (11) which may impair the contractility of the myocardium and leads to systolic dysfunction.

Several studies have showed that, LV diastolic dysfunction represent the earliest pre-clinical manifestation of myocardial involvement in diabetes, preceding systolic dysfunction (12,13). Moreover, the dysfunction can be observed before the development of diabetes (9) suggesting that it is not only a complication of diabetes but rather a coexisting condition. Bajraktari and cols. showed that insulin resistance is an independent correlate of LV diastolic dysfunction in subjects with IGT and type 2 DM. In this study, a significantly greater proprotion of patients with echocardiographic evidence of LV diastolic dysfunction was observed in subject with IGT and DM compared with subjects with normal glucose tolerance (14). Therefore, a comprehensive assessment of cardiovascular changes in prediabetic patients should be always kept in mind and not overlooked. We therefore studied normotensive prediabetic patients in whom the prevalence of hyperglycemia and impaired glucose metabolism is high to hypothesed that the prediabetes is associated with subclinical LV systolic and diastolic function. At the end of the study, we have supported our hypothesis.

We used TDE in addition to conventional echocardiographic techniques to evaluate LV function. TDE is used in varying cardiac conditions with validation as a marker of LV systolic dysfunction (15,16) and diastolic dysfunction (17,18). It is useful in the evaluation of coronary artery disease (19) and has prognostic implications (20,21). In addition, it is more sensitive than conventional echocardiography for detecting early myocardial changes in primary (cardiomyopaties) and secondary (ischemia) myocardial disorders (20,22). Routine echocardiographic assessment of regional left ventricular (LV) wall motion is subjective because it is determined by visual determination of endocardial excursion and wall thickening. TDE offers the promise of an objective measure to quantify regional and global LV function through the assessment of myocardial velocity data. Also, conventional PW Doppler measurements may be affected by preload, afterload, heart rate, inhalation and exhalation. In addition, the pseudonormalized form may complicate the diagnosis. Hence, TDE is useful for screening and detection of subclinical myocardial dysfunction, and for evaluating the efficacy of therapeutic interventions (22,23). TDE measurements are not affected from limiting variables of conventional echocardiographic methods such as blood pressure, ventricular geometry and loading conditions.

To evaluate myocardial systolic function, we have utilized the Sm and MPI parameters of TDE. Sm velocity mesures longitudinal LV contraction and is surrogate of LV systolic function. It demonstrated good correlation with LV ejection fraction. Em velocity is a measure of LV relaxation in early diastole and is relatively load independent (24). The ratio of E/Em correlates well with LV end diastolic pressure or pulmonary capillary wedge pressure (25). LA dilatation reflects chronic and long-standing pressure overload on left ventricle. It’s increased dimensions and reduction in Em velocity are demonstrated to be the strongest predictors for cardiovascular mortality (26). MPI has been widely used to quantitatively assess myocardial performance (27). It is more reflective of overall cardiac function than systolic or diastolic function alone in both ventricles (28,29). MPI has been studied in several other cardiac disorders including diabetes, myocardial infarction,hypertension and heart failure and found to predict both worsened morbidity and mortality (30-35).

In the present study, the significant decrease in LV Em and Em/Am ratio and increased E/Em ratio among prediabetic subjects compared to controls may imply subclinical phase of LV diastolic dysfunction. Furthermore, the significant increase in MPI and decreased Sm velocity may imply subclinical LV systolic dysfunction. Our results were consistent with the results of a recent study performed by Ceyhan and cols. (36). Using tissue Doppler and strain/strain rate echocardiography, they showed that LV longitudinal systolic and diastolic function were impaired in both normotensive diabetic and prediabetic patients. In another study, the prevalence of LV diastolic dysfunction was detected higher in patients with IGT as well as in those with newly detected and known DM but not in those with IFG. In this study, after adjusting for established risk factors, IGT but not IFG, was detected to be a predictor of LV diastolic dysfunction (37). However, in our study, the prevalence of LV diastolic dysfunction was higher in both IFG and IGT patients. This can be due to the higher number of IFG patients (52) included in our study compared to small number (18) of IFG patients in this study.

### Limitations of the study

There are some limitations of our study. First, the coronary artery disease was ruled out based on the history, physical examination, electrocardiography and echocardiography. The more precise imaging modalities like myocardial perfusion scintigraphy, conventional angiography or computed tomography angiography were not performed. Therefore, there may be subjects with CAD in our study population. Second, the relatively small number of patients included in this study prevents the generalization of these results to all prediabetic patients and made difficult to perform subgroup analysis. Third, tissue Doppler echocardiography measurements are angle dependent and therefore the values obtained through this method may be influenced by that limitation. To overcome this, our values were evaluated by 2 investigator. Third, although LA volume is a more precise indicator of chronic diastolic dysfunction, we have utilized LA diameter. Fourth, global longitudinal strain (GLS) measured by 2-dimensional longitudinal speckle-tracking echocardiography may be a more sensitive measure of left ventricular function than conventional LV ejection fraction (EF), and more useful than MPI.

In conclusion, our data demonstrated that there is both systolic and diastolic left ventricular dysfunction in prediabetic patients. TDE is especially important in detecting the early subclinical LV systolic and diastolic dysfunction at this group of patients and its subgroups. For that reason, utilization of TDE during conventional echocardiographic measurements of prediabetic patients may have prognostic significance. These early abnormalities observed in LV function may explain the increased morbidity and mortality seen in patients with preDM. However, the clinical significance of our findings remains to be determined with larger scale studies.
